# Supplementary material for: Are carbon emissions trading and green financial instruments synergistic? -Comprehensive quantitative research based on content analysis
Source: PLoS One. 2024 Mar 7;19(3):e0298601. doi: 10.1371/journal.pone.0298601 (PMC10919601; doi:10.1371/journal.pone.0298601)
Supplement: S1 Data — (ZIP) [file pone.0298601.s001.zip › Data/地级市Z_F工作报告.zip!/║╙▒▒/▓╫╓▌╩╨/└┤╫╘╣1⁄2╓┌║┼í╛┬φ┐╦╩2╛▌═°í┐_2019.docx]

各位代表：

现在，我代表沧州市人民政府向大会作工作报告，请予审议，并请各位政协委员和列席会议的同志提出意见。

一、2018年工作回顾

刚刚过去的一年，是我市发展进程中不平凡的一年，也是沧州砥砺前行、负重奋进的一年。在市委的坚强领导下，我们坚持以习近平新时代中国特色社会主义思想为指导，坚决贯彻落实省委、省政府和市委重大决策部署，抢抓机遇、迎难而上，全市经济社会保持平稳健康发展态势。预计全市生产总值增长6.4%，一般公共预算收入增长10%，规上工业增加值增长4.1%，固定资产投资增长5.3%，社会消费品零售总额增长9%，城乡居民人均可支配收入分别增长8%和8.5%。

（一）协同发展纵深推进，对接成效持续显现。全方位对接京津、承接转移，与京津合作产业项目1100余个，协议总投资5714亿元。北京现代沧州工厂累计产销整车31万辆，北汽新能源汽车黄骅基地实现量产；北京生物医药产业园签约入驻药企137家，总投资438亿元；东塑明珠商贸城、明珠服饰产业特色小镇整体承接北京大红门、动批等服装商贸企业，8000多家商户无一回流，中央电视台《新闻联播》进行专题报道。综合交通网络建设加快推进，石衡沧港城际铁路通过核准，曲港高速（曲阳至肃宁段）建成通车，中捷通用机场正式投用。全力支持服务雄安新区规划建设，黄骅港作为雄安新区出海口写入《河北雄安新区规划纲要》。我市协同发展工作被中央协同办考察评估组誉为承接京津产业疏解“沧州现象”。

（二）沿海经济支撑强劲，渤海新区活力迸发。启动实施总投资585亿元的20个专业码头和23个港区重点基础设施项目，20万吨级矿石码头正式开放。黄骅港全年完成吞吐量2.88亿吨，集装箱71.7万标箱，分别增长6.5%和9.7%。新开工亿元以上产业项目315个，鑫海四期、三和重工装备制造等114个项目竣工投产。恒大文化旅游城、碧桂园生态城、沧海文化风景区等一批文旅康养项目加快推进，扮靓绿色新区。预计渤海新区固定资产投资、规上工业增加值分别增长11.6%和9.3%，高于全市平均水平6.3和5.2个百分点，龙头带动作用进一步显现。

（三）新兴产业蓄势发力，转型升级步伐加快。组织实施“六个一”工程，做大做强“18+7”特色产业集群，全市工业技改投资增长7.5%，33个项目列入2018年度省工业新产品新技术开发名录。全市新增规模以上工业企业158家。下大力壮大“六大新动能”、培育“五大新引擎”，汽车及零部件、清洁能源实现增加值分别增长44.5%和52.7%，生物医药、主题旅游投资增速分别为53.9%和59%；华北石化千万吨炼油项目正式投产，年销售收入可达640亿元；京津冀再制造产业技术研究院建成投用，华为云计算中心机房主体完工。全市新动能、新引擎涵盖入统企业近千家，增加值对全市GDP贡献率达35.1%。创新要素持续活跃，新增省级技术创新中心8家、院士工作站11家，总数分别达到29家和30家；新增高新技术企业150家、科技型中小企业1618家。服务业投资增长14.9%，高于全市固投增速9.6个百分点。

（四）城乡发展协调并进，环境面貌有效改观。围绕增强综合承载力、辐射带动力，统筹推进城乡提质提速发展。中心城区新建改建公园游园20个，南北绕城项目实现阶段性通车；高标准完成197个老旧小区改造，工程进度居全省首位；中心城区11个菜市场升级改造工程全部完成；占地1200亩的大运河城市区生态修复及环境卫生整治工程展示区建成开放，真正做到还运河于人民，让老人能散步、青年能运动、孩子能嬉戏。强力推进城乡环境卫生“攻坚月”“提升月”活动，3531个村（居）实现城乡环卫一体化。实施“一区三边”违法建设整治行动，拆除整改“大棚房”619栋、违法违规建筑面积119万平方米。加大房地产遗留问题整治力度，办结分户登记4.47万套，办证率70.3%。智慧沧州手机APP上线运行。县城建设步伐加快，任丘入围“全国新型城镇化质量百强县市”，黄骅获评“四好农村路”全国示范县。我市顺利通过国家节水型城市验收和国家园林城市省级复查。农村土地承包经营权确权登记颁证工作基本完成；新启动“三区同建”示范区37个，新增市级现代农业园区15个，农村土地流转率、农业产业化率分别达到45.3%和66.5%。沧州（南皮）国家农业科技园区通过科技部验收。

（五）各项改革落地落实，开放优势巩固提升。紧跟中央和省委、市委部署抓好改革创新工作，全面启动并基本完成70家市属国有集体企业改革工作，“放管服”、开发区体制机制、市直事业单位公务用车制度改革扎实推进。市本级取消、下放行政权力497项，在全省率先建成市县乡村四级一体化在线服务平台。设立央企服务中心，建立“企业家直通车”制度，助推实体经济发展。全市新增市场主体8.8万户，达到43.3万户。积极推进政银企对接，投放风险缓释基金27.4亿元，节省企业融资成本9600余万元。沧州位列《中国城市政商关系排行榜》第14位。公安系统辅警队伍管理体制和农村交管两项改革走在全省前列。中心医院兼并重组市政府招待处工作基本结束。成功举办首届中国-中东欧中小企业合作论坛、中英绿色制造与再制造产业发展峰会，组织创业中华海外侨商沧州行、海归博士沧州创新创业行、百家大型农业企业走进沧州等系列招商推介活动，签约项目总投资1413.5亿元。河间获批“国家级外贸转型升级基地”。全市实际利用外资6.7亿美元、外贸进出口317.3亿元，分别增长7.8%和27.7%，其中出口增长22.2%，增幅全省第一。

（六）三大攻坚扎实开展，发展基础更加稳固。严控金融风险，依法稳妥有序处置滨海大宗问题，全面完成存量政府债务置换。有力应对百年不遇的台风暴雨侵袭，实现安全度汛。全力做好脱贫攻坚工作，7个贫困县全部摘帽，674个贫困村全部退出，在全省率先实现整体脱贫。扎实打好蓝天、碧水、净土三大保卫战，淘汰燃煤锅炉855台，完成气（电）代煤23.45万户，整治“散乱污”企业2244家，主城区烟花爆竹全面实现禁售禁放，全市PM2.5平均浓度下降10.6%，完成省定任务目标；认真落实河（湖）长制，强力推进水污染治理，完成老石碑河生态治理修复工程，近岸海域水质整体达到优良水平；全年完成造林51万亩，森林覆盖率达到31%。同时，严防环保“一刀切”，铸造、建材等六大重点行业实行差异化错峰生产。

（七）人民生活持续改善，社会事业全面进步。扎实推进22项重点民生工程。城镇新增就业10.9万人。棚户区改造开工1.1万套，改造农村危房2942户。新改扩建及完善提升公办幼儿园92所，创建标准化学校60所；全市提前两年全部通过县域义务教育基本均衡国家认定；各学段生均公用经费实现高标准全覆盖。在全省率先将外出农民工和外来就业创业人员全部纳入异地就医直接结算范围，黄骅市通过家庭医生管医保推进基层健康管理经验做法受到国务院通报表扬，沧州传染病医院列为国家级现代医院管理制度试点。运动休闲城市建设稳步推进，新建改造体育设施123处，4座市级体育场馆如期竣工，600个行政村建成农民健身工程。国家公共文化服务体系示范区顺利通过验收，“文化干细胞”培育工程和“城市书吧”模式全省推广。圆满承办河北省第十届少数民族传统体育运动会，成功举办第十届中国•沧州国际武术节和2018年沧州市旅游产业发展大会，展现“千年杂技梦•百里运河图”独特神韵。安全生产形势总体平稳，扫黑除恶专项斗争取得积极成果。退役军人服务管理工作扎实有效，国防动员和双拥共建深入开展。民族宗教、外事侨务、人民防空、史志档案、援藏援疆、妇女儿童、老龄、残疾人、红十字会等工作都取得了新成绩。

（八）政府建设不断加强，工作作风持续转变。认真贯彻落实党的十九大精神，旗帜鲜明讲政治，树牢“四个意识”，坚定“四个自信”，坚决维护以习近平同志为核心的党中央权威和集中统一领导。大力推进政府系统党的建设，坚决落实中央巡视、国务院大督查和省委巡视反馈问题整改，持之以恒正风肃纪，扎实开展“一问责八清理”、基层“微腐败”专项整治行动，政风行风持续好转。严格执行“日清日结”和“黄牌警告”制度，扎实推进“双创双服”活动，全市政府系统机关办事效率显著提升。办理人大代表建议和政协提案672件，办复率均为100%。

各位代表，过去的一年，我们面临的形势比预计的更严峻，遇到的困难比原想的更复杂，取得的成绩更为难能可贵。一年来，沧州的发展受到党中央、国务院和省委、省政府高度关注，中国-中东欧（沧州）中小企业合作区建设写入《中国-中东欧国家合作索非亚纲要》，省委、省政府主要领导先后莅沧考察指导工作，明确了沧州“一港双城三带四区”发展格局。一年来，面对前所未有的经济下行压力，我们沉着应对、综合施策、精准发力，主要指标止跌回升，经济运行呈现稳中向好、稳中有进态势；面对亟待破解的产业转型升级发展命题，我们坚持政策扶持引导、激活企业内生动力并举，做优存量、做强增量齐抓，推进实施工业转型升级、战略性新兴产业发展、科技创新等系列三年行动计划，全面提升产业综合实力，沧州在福布斯公布的“中国大陆最佳地级城市30强”中列19位，在2018中国城市产业竞争力百强榜中列55位；面对人民对美好生活的新期待，我们坚持抓重点补短板强弱项，以使命初心回应解决老百姓关心的身边事、具体事，脱贫攻坚、运河改造、拆迁拆违、房地产遗留问题整治等工作取得突破性进展；面对艰巨繁重的改革发展任务，各级各部门以迎难而上、敢为人先的干事激情，主动为全市发展担担子、做贡献，渤海新区主要经济指标连续多年领跑全市，黄骅、任丘、河间、泊头等县（市）多项创新性举措和经验做法在全省全国复制推广。这些成绩的取得，是认真落实省委、省政府决策部署和对沧州具体要求的结果，是市委统揽全局、坚强领导的结果，是市人大、市政协有力监督、大力支持的结果，是全市人民凝心聚力、共同奋斗的结果。在此，我代表沧州市人民政府，向全市人民，向人大代表、政协委员，向各民主党派、工商联、人民团体和各界人士，向驻沧解放军、武警官兵和政法干警，向所有关心支持沧州发展的同志们、朋友们，致以崇高的敬意和衷心的感谢！

各位代表，安不忘危，稳不忘忧。当前沧州发展面临的主要困难和问题是：经济承压运行，高质量有效投资后劲不足；科技创新能力较弱，发展动能转换还不够快；城镇化率偏低，城市管理水平有待进一步提升；生态环境治理任重道远，空气质量持续改善的压力依然很大；民生领域还有不少短板，防风险守底线任务繁重；一些部门和工作人员标准不高、作风不实、执行力不强等现象不同程度存在。对这些问题我们一定高度重视，下大力解决。

二、2019年目标任务和总体要求

2019年是新中国成立70周年，是全面建成小康社会关键之年。从宏观形势看，尽管国内经济运行稳中有变、变中有忧，但我国发展仍处于并将长期处于重要战略机遇期，党中央审时度势、把航定向，持续加大宏观调控力度，我国经济一定会稳定运行在合理区间。从我市自身情况看，虽然短期内发展会受到外部环境不确定性、复杂性因素影响，经济面临一定下行压力，但历经40年改革开放，沧州在建设经济强省、服务协同发展大局中的地位和作用日益增强，城市的影响力、辐射力和美誉度持续提升。推进创新发展、绿色发展、高质量发展，我们有坚强有力的政治引领、有千载难逢的重大机遇、有经年累积的产业基础，这些都为沧州提供了巨大发展势能和重大战略支撑。只要我们深入贯彻执行中央和省决策部署，切实把市委既定工作思路坚持好、落实好，就一定能应势而动、顺势而为、乘势而上，走出一条具有沧州特色、沧州优势、沧州高度的新路子，开创沧州发展的美好未来。

我们要坚定不移抢抓“三大机遇”，这是沧州实现高质量发展必须借助的强劲东风。京津冀协同发展、雄安新区规划建设、共建“一带一路”三大国家战略机遇在沧州交汇叠加、集聚释放，这在我市历史上前所未有。机不可失，时不再来。我们必须紧跟时代潮流，以时不我待的紧迫感和责任感，牢牢抓住千载难逢的历史性窗口期和战略性机遇期，用历史眼光观大势，用战略思维谋全局，以沧州之“力”助推协同发展，以沧州之“为”激荡开放大潮。

我们要坚定不移发挥“三大优势”，这是沧州实现高质量发展必须依靠的基础条件。沧州沿海有港，亿吨级大港——黄骅港已成为亚欧大陆桥新通道桥头堡；沧州区位优越，是京津冀一小时经济圈重要成员；沧州拥有53万亩建设用地和200余万亩未利用地，是发展临港产业的“黄金宝地”。我们必须围绕发挥优势做文章，深挖内部潜力，变资源为资产，通过聚合、裂变，把比较优势转化为发展胜势、汇聚成经济强势，在日趋激烈的区域竞争中赢得更大主动。

我们要坚定不移打好“三大攻坚战”，这是沧州实现高质量发展必须跨越的险阻关口。防范化解重大风险、精准脱贫、污染防治三大攻坚战打得好不好，直接关系到全面建成小康社会质量高不高。我们必须牢固树立以人民为中心的发展思想，坚持问题导向、实践导向、效果导向，以更加自觉的历史担当、雷厉风行的治理行动和坚韧不拔的顽强意志，攻下最难的堡垒、啃下最硬的骨头，全力补短板、强弱项，为沧州率先实现全面建成小康社会目标奠定坚实基础。

我们要坚定不移抓好“四件大事”，这是沧州实现高质量发展必须筑牢的四梁八柱。沿海经济带建设、产业转型升级、改善人居环境和推进乡村振兴，事关全局、影响长远，抓好这“四件大事”，是市委市政府立足省情、结合市情作出的战略部署。我们必须把既定目标要求，细化到每项任务、量化到每个节点，层层分解压责任、不折不扣抓落实，努力为全市经济社会发展强根基、健筋骨，铺就行稳致远的道路坦途。

我们要坚定不移壮大“六大新动能”、培育“五大新引擎”，这是沧州实现高质量发展必须连通的隧道桥梁。新时代的发展，是追求更高质量、更可持续的发展，是沧州摆脱传统路径依赖、实现蜕变提升的转型发展。我们必须坚持质量第一、效益优先，通过“六大新动能”和“五大新引擎”的加速成长，调结构、转方式，构建特色现代产业体系，真正让新经济、新业态、新模式在沧州遍地开花、结出硕果。

今年政府工作的总体要求是：深入学习贯彻习近平新时代中国特色社会主义思想和党的十九大精神，认真落实中央经济工作会议、省委九届八次全会、市委九届五次全会部署，坚持稳中求进工作总基调，坚持新发展理念，坚持推动高质量发展，坚持以供给侧结构性改革为主线，坚持深化改革开放，紧紧围绕“沿海率先发展创新示范区、环渤海地区重要的现代化工业城市”功能定位，构建“一港双城三带四区”发展格局，抢抓三大机遇，发挥三大优势，继续打好“三大攻坚战”，抓好“四件大事”，壮大“六大新动能”、培育“五大新引擎”，统筹推进稳增长、促改革、调结构、惠民生、防风险工作，进一步稳就业、稳金融、稳外贸、稳外资、稳投资、稳预期，推动沧州创新发展、绿色发展、高质量发展走在全省前列，加快建设新时代创新驱动经济强市、生态宜居美丽沧州，为全面建成小康社会收官打下决定性基础，以优异成绩庆祝中华人民共和国成立70周年。

今年经济社会发展的主要预期目标是：全市生产总值增长7.5%，一般公共预算收入增长7.5%以上，规模以上工业增加值增长6%，固定资产投资增长7%，社会消费品零售总额增长10%，城乡居民人均可支配收入分别增长8.5%和9%；PM2.5平均浓度下降幅度、主要污染物减排总量、环境质量控制等完成省定任务。上述目标的设定，既遵循了中央“六稳”工作要求，又与我市“十三五”规划目标相衔接，综合考虑了外部经济环境、我市发展形势和各方面支撑条件，是一个实事求是的目标，也是一个积极进取的目标。

在实际工作中，需要把握好以下几个方面：一是牢牢把握稳中求进这个工作总基调。坚持以经济建设为中心不动摇，切实处理好“稳”和“进”的辩证统一关系，确保在经济增长、民生底线、风险防控上稳得住，在转换动能、调优结构、提高效益上进得好，以进促稳、以稳应变。二是牢牢把握供给侧结构性改革这条主线。坚持运用市场化、法治化手段，在贯彻“巩固、增强、提升、畅通”八字方针上下功夫，以深入实施系列三年行动计划为抓手，巩固“三去一降一补”成果，增强微观主体活力，提升产业链水平，畅通经济循环。三是牢牢把握改革开放这一动力。坚持深化市场化改革，全面落实中央、省改革部署，着力健全与高质量发展相适应的体制机制，推进各项改革走深走实；坚持高水平扩大开放，搭建平台、提升能级，在更大范围、更广领域、更高层次参与全球资源配置和国际市场竞争。四是牢牢把握增进人民福祉这个根本。坚持普惠均衡、共建共享，兼顾长远、分步实施，让全市人民有更多实实在在的获得感、幸福感、安全感。

三、2019年重点工作

统筹推进今年经济社会发展各项工作，必须突出重点、把握关键，切实做到齐头并进不落项、主次轻重要分明。重点做好以下十个方面：

（一）聚焦政策引领、要素保障，在促进经济持续平稳发展上再发力

精准扩大有效投资。突出项目这个核心，创新理念抓项目、招商引资抓项目、补齐短板抓项目，全年实施重大战略支撑项目50个、省重点项目36个、市重点项目200个，确保北京利和知信80万吨高性能树脂、河北科光宽禁带化合物半导体等200个项目开工建设，达力普石油装备、中泊防爆工具及铜制品深加工等100个项目竣工投产，完成投资1200亿元以上。强化调度督导，大力实行领导分包、观摩考评、集中开工、巡查督导、跟踪问效“五项机制”，统筹项目用地用海、市政配套、环评审批，坚决防止因要素保障不到位出现“半拉子”工程。运用好产业引导基金、地方政府债券等方式，撬动更多社会资本投入城市基础设施、农业农村设施建设，激发社会资本投资活力。

深入挖掘消费潜力。适应服务消费增速加快、消费结构升级新趋势，大力发展金融保险、楼宇经济、会展经济等生产性服务业和医疗康养、教育培训、旅游健身等生活性服务业，在中高端消费、绿色低碳、共享经济等领域培育新的增长点。深入推进“增品种、提品质、创品牌”行动，增加高品质商品供给。加快建设一批高端商业综合体、商业街区，进一步活跃城市夜经济。开展电子商务进农村综合示范县建设，支持阿里巴巴沧州产业带发展，创建省级电子商务示范基地1个、示范企业3个。

持续增强出口拉动。落实和完善鼓励出口政策，进一步拓展对外贸易空间。实施外贸优势品牌认定行动计划，打造11个县域特色产业外贸基地。支持黄骅市创建省级跨境电子商务园区，布局建设5个海外仓和特色产品展销中心，积极创建省级服务外包示范市。开展外贸企业精准帮扶行动，培育3家外贸出口领军企业，提升贸易出口商品附加值，推动加工贸易产业链升级，力争外贸进出口总值增长7%以上。

做优做强实体经济。落实国家更大规模减税降费政策，对小微企业和科技型初创企业实施普惠性税收减免，降低用能和物流成本，清理和规范各种附加收费。深入实施“万企转型”行动和“双百”工程，扶优做强骨干企业。推动“个转企、小升规”，培育“专精特新”中小企业10家，新增规上工业企业80家以上。引导银行向企业发放中长期贷款，落实降低小微企业融资担保成本的奖补机制，积极帮助企业拓宽融资渠道，进一步放大中小企业风险缓释基金“活水”作用，着力破解贷款难、担保难、融资贵等问题。大力实施质量强市和标准化战略，主导或参与制（修）订国家、行业标准10项以上，培育一批在国内有影响力的标准领跑企业。实体经济是业之母、财之源、民之本，必须坚定不移鼓励支持、发展壮大。

（二）聚焦对接合作、功能疏解，在深度融入协同发展大局上再发力

扩展产业对接。加强与北京中关村、丰台区及大型央企民企合作，引进一批新产品新技术的研发、生产、配套服务项目。加快落实与天津滨海新区、东丽区、红桥区战略合作协议，力争在港口建设、产业发展等方面形成一批合作成果。抢抓雄安新区即将进入大规模开发建设阶段的机遇，紧盯产业布局和政策导向，实现无缝衔接、错位发展，打造“雄安新区出海口和清洁能源供应基地、制造业协作基地、科技成果研发转化基地”。

搞好平台对接。围绕“接得住、接得好、接得牢”，按照合作共建、产业互动、协同发展原则，着力打造沧州现代产业基地、渤海新区新型工业化基地、任丘石化产业基地三大对接平台，加快推进京南国家科技成果转移转化、沧州产业协同发展、京津冀产业转移现代制造业改革3个示范区建设，全力抓好沧州高新区高新技术创新中心、中心城区总部经济转移基地等10个承接北京非首都功能转移“微中心”。

提速功能对接。进一步健全完善要素保障、配套服务等九大机制，加强与北京企业、高校、科研院所的对接，完善创新链和产业链配套，形成北京研发、沧州转化、沧州制造的合作新模式。积极承接京津汽车、医药产业和服装批发市场转移，制定出台更加优惠的政策和措施，全力做好教育、住房、医疗等配套服务，确保商户落得下、留得住、发展得好。

加快交通对接。积极落实《推进河北省现代化综合交通运输体系建设三年行动方案》，重点抓好津沧、雄沧港、环渤海城际铁路和雄商高铁、任沧港快速路，以及京德、曲港高速公路等重大项目前期工作，力争石衡沧港城际铁路、邯港高速公路年内开工，推动沧州与京津、雄安、省会的互联互通，建设“轨道上的京津冀”重要交通枢纽。

（三）聚焦港城融合、产城共兴，在加快沿海经济带建设上再发力

打造国际一流综合大港。着眼建设现代化综合服务港、国际贸易港和“一带一路”重要枢纽，深入实施“港口建设三年行动计划”，全力抓好河钢通用散货、泰地液体化工等总投资360亿元的20个码头群建设。进一步拓展丰富航线，加快口岸查验设施建设，增强通关运营能力，力争全年吞吐量达到2.9亿吨、集装箱75万标箱以上。深入实施“九路三互通一出口”工程，打通液体散货管廊运输网络，大力发展海铁联运、多式联运，全面提升港口面向华北、东北、西北地区的吸引辐射功能。加快推进黄骅港与天津港股权合作，全力申报国家自由贸易港区。

打造现代临港产业体系。优化提升园区功能布局，大力推行“一区多园”管理模式，实现错位互补、竞相发展。加快构建以先进制造业为主导的临港产业体系，重点抓好沧州大化PC、嘉好粮油二期、大唐电厂等409个亿元以上产业项目，确保北交大海滨轨道交通综合研发实验基地、广祥制药等90个项目竣工投产，生产总值和固投增速达到两位数以上。大力发展海洋经济，加快推进巴安水务、阿科凌、国华沧东等海水淡化项目建设，争创国家海洋经济发展示范区。

打造区域对外开放高地。按照“国际标准、欧洲特色”的规划思路，加快推进中国-中东欧（沧州）中小企业合作区建设，启动实施“五大先导工程”和“十大重点产业项目”，尽快“走热”中东欧商贸线、友谊线；精心筹办“第二届中国-中东欧中小企业合作论坛”，将其打造成具有国际影响力的常态化品牌活动。加强与海关总署等国家有关部委的沟通对接，确保黄骅港综合保税区申报成功、封关运行。放大临港经济技术开发区引领效应，加快推进港城区等园区托管工作。

打造绿色生态宜居新区。统筹管理海域资源和环境容量，严把闲置用地处置关、土地节约集约关，严控高污染、高耗能、高耗水项目。加快黄骅港大宗货物“公转铁”步伐，大力推广船用岸电等清洁能源使用。稳步推进黄骅新城建设，启动实施“七河一连通一治理”工程，重点抓好127个城市基础配套项目，以及中东欧十六国商务中心、尼特拉风情小镇等文旅项目。推动黄骅、中捷、南大港相向融合对接、彰显地域特色，打造高品质现代化滨海新城。

（四）聚焦产业塑造、动能转换，在构建现代化产业体系上再发力

做强特色产业集群。围绕落实工业转型升级三年行动计划、做大做强“18+7”特色产业集群，深入实施“六个一”工程，进一步提升园区承载力、展会影响力，发挥好智能改造计划的引领作用、产业提升基金的助推作用、研发中心的促进作用、行业协会的保障作用，推动东光包装机械等15个产业集群加快向百亿级规模迈进。完善工业设计政策体系，积极推进市县两级工业设计创新中心建设。支持河间、泊头开展工业设计应用示范，在产品研发、外观设计、产品包装等环节植入工业设计元素。大力实施制造业质量提升等10个专项行动，推动先进制造业与现代服务业、军工与民企融合发展，支持孟村弯头管件、泊头铸造、盐山管道装备制造等产业园区争创省级、国家级“知名品牌创建示范区”。

壮大“六大新动能”。（1）汽车及零部件制造。发挥北京现代、北汽集团等龙头企业的带动作用，积极引进京津高端配件企业，争取北京现代一二工厂产能转移，加快北京现代新能源汽车、龙创新能源汽车产业园建设，打造华北地区重要汽车全产业链生产基地，力争2020年实现产值1400亿元。（2）绿色化工。依托中石油、中海油、中石化等龙头企业，积极推进下游精细化工及轻纺产业发展，重点抓好百万吨乙烯、旭阳化工己内酰胺等项目建设，力争2020年实现产值3500亿元。（3）生物医药。依托北京和天津生物医药产业园、石家庄四药，积极承接一批现代微生物药物、基因工程药物、新型制剂、现代中药等关键技术成果转化和产业转移，重点抓好北京协和药厂、天成药业抗肿瘤药等项目建设，加快打造千亿级产业集群。（4）主题旅游。依托沧州自然人文特色元素，加快恒大文化旅游城、碧桂园文旅健康生态城等项目建设，推进吴桥杂技文化产业园、东光铁佛寺、河间府署等景区提档升级，打造大运河文化体验带、渤海湾休闲度假带、沿雄安生态养生带。办好2019年沧州市旅游产业发展大会，擦亮“运河古郡•渤海明珠”旅游品牌，力争旅游业总收入增长20%以上。（5）时尚服装服饰。抢抓北京服装服饰产业疏解机遇，以明珠国际服饰产业特色小镇、华斯裘皮特色小镇等项目为龙头，整合提升原有纺织、皮毛等产业，加快构建服装设计、生产、批发为一体的全产业链新业态。（6）现代物流。做大做强渤海新区、肃宁、沧东3个省级物流产业聚集区，加快推进新华区传化智能公路港、河间海尔智慧物流园等项目建设，打造环渤海地区重要港口物流产业基地和京津冀城市群重要物流产业支撑基地。

培育“五大新引擎”。（1）激光及智能制造。依托激光加工国家工程研究中心京津冀区域中心，大力发展激光研发应用和智能装备制造产业，突出抓好华工森茂特、领创科技、沃克曼等龙头企业，打造京津冀区域激光加工技术示范和推广基地。（2）先进再制造。依托京津冀再制造产业示范基地，以再制造院士工作站、中英再制造工业园为平台，加快发展汽车零部件、石油钻采设备等领域再制造技术的研发应用，打造国内一流的再制造高度聚集发展区。（3）电子信息及应用。加快推进华为云计算数据中心、智慧沧州“城市大脑”、苏州纳米研究院、渤海新区微电子产业园等项目建设，加强与阿里巴巴、九次方、腾讯等信息产业巨头合作，大力发展数字经济、智慧产业，打造电子信息及应用产业示范园区。（4）航空航天技术。以中捷通用航空产业园为龙头，加快沧州开发区军民融合航天科创小镇、吴桥驰宇通航产业园等项目建设，打造集飞机培训、航空展示、制造维修、研发应用于一体的航空航天产业基地。（5）清洁能源。依托沿海地区可再生资源和地质结构的优势，大力发展太阳能、风能、核能等新型能源，建设多元化清洁能源供应体系，积极推进海兴核电和核燃料产业园前期工作。

（五）聚焦扩容提质、均衡协调，在推动新型城镇化建设上再发力

实施空间拓展工程。围绕融入京津冀世界级城市群，对标对表雄安新区规划设计先进理念，按照“多规合一”要求和“内疏外聚”思路，高标准编制《沧州市城市总体规划（2018-2035）》，做好国土空间规划基础工作，有序推进行政区划优化调整。着眼中心城区东西部均衡发展，支持新华区“东扩南进”、拉大框架，运河区完善功能、丰富内涵；推进东向与沧州经济开发区和沧东开发区、北向与青县的道路互通、组团式发展，形成“东西并进、南北均衡”的整体空间架构，提高城市首位度和发展能级。进一步加大中心城区土地收储出让力度，确保年内收储商住用地5000亩以上，为城市发展提供有力土地保障。

实施道路畅通工程。积极谋划推进城市大外环前期工作。南北绕城项目9月底前全线竣工通车；高标准实施海河路提升改造工程，打造沧州东西向又一条“迎宾大道”；大力推进全长7.3公里的104国道改扩建工程，缓解东部城区交通压力；永济路年内建成通车，全线实现雨污分流。优化主城区路网结构，修建杭州路、百川路等10条市政道路，进一步提高市民出行便利性。

实施功能提升工程。按照现代化、国际化城市标准，以运动休闲城市、卫生城市、智慧城市、海绵城市、森林城市、文明城市“六城联创”为抓手，实施一批重点城建工程。加快大运河文化带建设，编制实施保护传承利用规划纲要及城市区段实施规划，对沧州境内215公里运河统筹谋划、全域打造，确保“大化•1973”文化主题园、工人文化宫年内开工，高标准打造“中国大运河文化重要承载地、河北省城市生态休闲走廊示范区、沧州市城市重要标志”。启动市殡仪馆外迁和公墓建设工程。抓好吉林大道、千童北大道等21条道路绿化提升，新改建街角游园25个，力争滨河公园全线贯通，名人植物园二期建成开放。高标准完成258个老旧小区改造，让更多小区旧貌换新颜。

实施治理提质工程。积极推动大数据、云计算、人工智能在城市治理、政务服务、电子商务等领域的应用，让居民享受智慧城市建设带来的便利生活。大力实施城乡洁净工程，中心城区高速围合区内全面实现环卫作业市场化，机械化清扫率达到95%以上。以违章建筑私搭乱建、车辆乱停乱放、牌匾广告乱贴乱挂、流动摊点乱摆乱卖、建筑围挡乱立乱设“五乱共治”为抓手，全面提升城市管理水平。新建改建厕所110座、谋划建设和升级改造停车场11个、全面消除黑臭水体，让城市容貌更整洁、更有序，市民生活更舒心、更美好。

实施县城和小城镇建设提档工程。推进县城改造提升，加快乡改镇、镇改街、村改居步伐，促进农村人口向城镇有序转移，力争全市常住人口城镇化率提高2个百分点。围绕实现城乡垃圾处理无害化，启动实施任丘、献县等7个生活垃圾焚烧发电厂，规划建设14个建筑垃圾消纳场。鼓励有条件的县城争创“洁净城市”“卫生县城”“文明县城”，力争任丘成功创建国家园林城市。培育壮大县域综合实力，争取更多县（市）进入全省30强、全国“百强县”。按照“特色小镇镶运河”理念，积极推进沧县旧州古文化城、青县红木小镇等特色小镇建设。

（六）聚焦大众创业、万众创新，在全面提升科技创新能力上再发力

培育壮大创新主体。落实科技创新三年行动计划，实施高新技术企业后备培育和科技型中小企业扩量提质工程，引导优势科技资源和创新服务向企业聚集，力争全市新增高新技术企业100家，科技型中小企业总数超万家。建立以财政、企业、金融、市场为支撑的多元化科技创新投入体系，大幅提高全社会研发经费投入占GDP比重。实施国家、省级重大科技项目50项，努力在关键性核心技术研发上实现新突破。

积极打造创新平台。鼓励河北工大•新华科技申报国家科技企业孵化器，支持南开大学（渤海新区）绿色化工研究院、北科院博士后工作站（黄骅）培养基地建设，年内新增省级以上技术创新中心、众创空间、星创天地10个，新建院士工作站、重点实验室5家。完善沧州科技大市场功能，推动科技资源开放整合与高效利用，全年技术合同成交额突破60亿元。

引进培养创新人才。启动“人才兴沧”工程，实施“千人计划”“外专百人计划”，推动“海归博士行”“院士行”等招才引智活动常态化，大力引进一批高层次创新创业团队。人才是第一资源，我们要广聚天下英才，让更多的“千里马”在沧州大地竞相奔驰，努力跑出创新之城的“加速度”。

（七）聚焦惠农兴业、环境提升，在深入实施乡村振兴战略上再发力

提升农业发展质量。深入实施农业供给侧结构性改革三年行动计划，大力发展科技农业、绿色农业、品牌农业、质量农业。严格粮食安全责任制，推动藏粮于地、藏粮于技，稳定提高粮食综合生产能力，不断增加优质绿色农产品供给。促进农村一二三产融合，培育一批农业产业化行业领军企业，重点抓好盐山中合•一同、黄骅新希望六和等50个超亿元农业项目，全市新增10个市级现代农业园区，产业化经营率达到67%以上。

改善乡村人居环境。推动实施河北省“千万工程”和农村人居环境整治三年行动，大力推进产业园区、生态园区、农村新型社区“三区同建”，新启动示范区30个以上，力争总数达到100个。新改建“四好农村路”400公里，改造农村公路危桥25座、生产桥68座、无害化卫生厕所6万座，县乡村全部实现垃圾城乡一体化处理，建设一批宜居宜业宜游的美丽乡村。

增强农村发展活力。完善农村承包地“三权分置”制度，稳慎推进宅基地制度改革。加快农村产权流转交易市场体系建设，实现县级交易中心全覆盖。积极推广土地流转“八大模式”，流转率达到50%以上。严格耕地保护，巩固“大棚房”专项治理成果，持续用力整治和防范，坚决遏制农地非农化。扎实推进农村新型经营主体建设，培养一批农业职业经理人、乡村工匠、文化能人。推进移风易俗，培育文明乡风、良好家风、淳朴民风。

（八）聚焦堵点难点、关键环节，在深化改革扩大开放上再发力

深化重点领域改革。深化国资国企改革，完善市级经营性国有资产集中统一监管体制机制，加快实现从管企业向管资本转变。深化事业单位分类改革，在加强清理规范的基础上，稳妥推进行政类、生产经营类和公益类事业单位改革。深化预算绩效管理，推进财政事权与支出责任划分改革，创新财政支持产业发展方式，增强财政资金的引导和放大作用。深化投融资体制改革，更加注重投资强度和质量，不断提高投资回报率、资源利用率、土地使用率、劳动生产率。深化统计管理体制改革，做好第四次全国经济普查。统筹推进教育、科技、文化、卫生等领域改革，充分释放改革红利。

构建全面开放格局。坚持“走出去”和“引进来”相结合， 不断拓展开放的深度和广度，加快构建海陆互动、内外双向开放新格局。一是扎好阵地营盘。深化开发区体制机制改革，加快“新九通一平”建设；以黄骅港综合保税区申报获批、沧州高新区创建国家级园区、提升中国-中东欧（沧州）中小企业合作区影响力为引爆点，带动全市省级以上开发区扩能升级，打造一批经济实力强、辐射带动强、支撑能力强的示范园区、国别园区。到2020年，全市开发区主营业务收入1000亿元以上的达到3家，生产总值和税收收入年均增长10%以上。二是搞好精准招商。坚持“以招商看发展论英雄”，分级健全招商引资工作机构，充实壮大招商队伍，完善招商企业目录和项目清单，加大领导招商、产业招商、社会化招商力度，力争全年洽谈引进亿元以上项目200个。三是办好推介活动。精心组织日韩、欧洲境外主题招商和北上广深系列招商活动，继续办好河间工艺玻璃、肃宁裘皮服装等大型展会，借助利用“5·18”廊洽会、“9·8”厦洽会等重要平台，积极对接世界500强、国内500强、大型央企和行业领军企业，吸引更多高端优质企业来沧投资发展。四是做好产能输出。深度融入“一带一路”建设，推动管道装备、机械制造等特色产能走出去，带动沧州企业嵌入全球产业链、价值链和创新链。

打造“六最”营商环境。深入开展“双创双服”活动，深化“一网一门一次”改革，推进“互联网+政务服务”，加大一体化在线服务平台推广使用力度，力争全流程网上办理和“最多跑一次”办理的事项比例不低于90%。推动“多证合一”“证照分离”和“联合审批”提速提效，工程建设项目审批时间压缩至100个工作日以内，全力打造河北领先、全国一流的“六最”营商环境，叫响“效率沧州”。

促进民营经济发展。认真落实促进民营经济发展的若干政策措施，努力消除民营企业在经营许可、经营运行、招投标方面的不公平待遇，让各类市场主体依法依规公平竞争。深化“企业家直通车”和领导干部包联制度，做到“有求必助、无事不扰、贴心服务”，打通为民营企业救急、解困、服务的“快车道”。民营企业家是社会财富的创造者，是我们的宝贵资源，要构建亲清新型政商关系，对企业家高看厚爱、真心呵护、倾力支持，让企业家切身感受到亲商、安商、富商的沧州温度。

（九）聚焦问题差距、弱项短板，在持续打好三大攻坚战上再发力

打好防范化解重大风险攻坚战。坚决防控金融风险，强化金融机构监管，严厉打击非法集资、融资等行为，稳妥做好滨海大宗问题处置工作。加强应急管理，提升防灾减灾救灾能力，持续深入开展安全生产大排查大整治，切实遏制重特大事故发生。深入推进扫黑除恶专项斗争，扎实做好房地产、政府债务、信访维稳等风险防控工作，为人民群众创造和谐稳定、安全有序的社会环境。

打好精准脱贫攻坚战。坚持脱贫不脱政策、摘帽不摘责任，继续实施产业扶贫攻坚和“百企帮百村”活动，统筹推进就业科技生态扶贫，增强贫困群众内生动力和自我发展能力。聚焦“两不愁三保障”，做好健康扶贫、教育扶贫、社会兜底扶贫，确保1.35万贫困人口稳定脱贫。建立精准防贫长效机制，扎实做好非贫困县脱贫攻坚，加大对贫困边缘户扶持力度，提高脱贫质量。

打好污染防治攻坚战。重点实施八大攻坚行动：一是实施冬季清洁取暖攻坚行动，坚持以气定改、以电定改，稳妥推进气（电）代煤工作。二是实施工业企业达标排放攻坚行动，对钢铁、火电、焦化等重点行业实施超低排放深度治理。三是实施“散乱污”企业整治攻坚行动，加大排查力度，做到“两断三清”，确保彻底整治到位。四是实施机动车污染管控攻坚行动，大力整治柴油货车超标排放，积极推进运输结构优化调整。五是实施扬尘污染防控攻坚行动，严格执行省定防治标准，坚决做到“六个百分之百”。六是实施水环境综合治理攻坚行动，严格落实“水10条”和河（湖）长制，突出重要河湖、水源地、近岸海域、地下水超采等重点领域和关键部位，坚持补水、蓄水、治水齐抓，实现标本兼治。七是实施土壤保护攻坚行动，全面落实“土10条”，狠抓土壤污染治理和生态保护修复。八是实施国土绿化攻坚行动，加快生态绿化带建设，植树造林30万亩，森林覆盖率达到32%。同时，深入抓好中央、省环保督察“回头看”反馈意见整改落实，以零容忍态度坚决打击环境违法行为。绿色是生命之源，我们要加强生态文明建设，齐心协力、共防共治，努力让沧州的天更蓝、水更清、环境更优美。

（十）聚焦群众关切、民生福祉，在创造美好生活上再发力

实施就业优先政策。完善就业创业资金管理办法，扎实做好下岗转岗职工、就业困难人员、退役军人等重点群体安置工作。支持农民工返乡创业，严厉打击恶意拖欠农民工工资行为。实施高校毕业生就业创业计划和基层成长计划，组织各类招聘服务活动500场以上，城镇新增就业6.4万人。

办好人民满意教育。以“立德树人”统领推进素质教育，推动义务教育均衡发展。新改扩建幼儿园20所，加大政府购买普惠性民办幼儿园学前教育服务力度，规范小区配套幼儿园建设使用。投资5亿元新改扩建中小学50所。启动中心城区第二轮教育资源布局调整，确保沧州市第二幼儿园分园、上海路小学、第十七中学建成投用，有效缓解“大班额”“择校热”等突出问题。深化义务教育学校县管校聘改革，到2020年全面推开。加快推进全市高中教育优质化、特色化发展，实施“双扩大”工程，深化职业教育产教融合、校企合作。支持高校优化专业设置，建设省内一流学科。

健全社保医保体系。深入实施全民参保计划，积极做好退役军人养老保险关系接续工作。全面推行以按病种付费为主的多元复合式医保支付方式，巩固完善异地就医住院费用直接结算，稳步提高基本医保和大病保险保障水平。完善最低生活保障、社会救助和法律援助等制度，健全农村留守儿童、妇女、老人关爱服务体系，落实好特困人员、残疾人、优抚对象等群体的兜底保障，城乡低保标准分别提高到7920元和4680元。

加快建设健康沧州。坚持“四医联动”，深化医药卫生体制改革，推广黄骅市“健联体”医改新模式和东光基层卫生院综合改革做法，推行分级诊疗和家庭医生签约服务，让群众在家门口享受良好医疗服务。实施中医药传承创新工程，加快沧州中心医院、沧州中西医结合医院分院区和任丘、肃宁等中医院项目建设。以做好贫困老年人集中供养为突破口，加强全市养老服务设施建设，新建养老院、社区居家养老服务中心（站）、精品互助幸福院56所。积极开展国家食品安全城市创建工作，让群众吃得更放心。

保障群众住有所居。合理有序供应土地，严厉打击违法建设、违规销售，保持房地产市场平稳健康发展。建立多主体供给、多渠道保障、租购并举的住房保障制度，对低保、低收入住房困难家庭加快实现应保尽保。全年开工棚户区改造5000套，完成797户“四类重点对象”农村危房改造任务。

繁荣文化体育事业。深入实施公共文化服务体系建设三年提升计划，大力开展“文化干细胞”和“十百千”优秀文化人才种子选手培育工程。启动沧州档案馆建设。整合杂技资源，培育领军人才，加快打造世界一流杂技艺术团体。精心组织好第十七届中国•吴桥国际杂技艺术节，力促杂技事业振兴发展。积极推进运动休闲城市建设，组织举办第十四届沧州市运动会，新建河北水利电力学院体育馆等体育设施80处，为500个行政村配建农民体育健身设施，全市新增体育场地面积71万平方米。

加强国防教育和国防后备力量建设，强化退役军人服务保障工作，维护军人军属合法权益，让军人成为全社会尊崇的职业。充分发挥工会、共青团、妇联等人民团体桥梁纽带作用。继续做好民族宗教、外事侨务、人民防空、气象地震、史志档案等工作。

各位代表，在多渠道多层次征求群众意见的基础上，按照尽力而为、量力而行的原则，今年谋划提出了24项民生实事，待会议投票确定后，我们将逐个制定专项实施方案，一项一项抓好落实，确保高标准、高质量完成。

四、全面加强政府自身建设

推动高质量发展、完成全面建成小康社会目标，需要我们有无比坚定的政治信念、担当担责的工作态度、攻坚克难的素质能力，这是党的要求，也是人民的重托。

（一）狠抓政治建设，强化思想引领。认真落实新时代党的建设总要求，深入学习贯彻习近平新时代中国特色社会主义思想，把践行“四个意识”和“两个维护”嵌入思想、融入灵魂，始终在政治立场、政治方向、政治原则、政治道路上同以习近平同志为核心的党中央保持高度一致。坚持党对一切工作的领导，围绕中央和省委、市委决策部署，一张蓝图干到底、一个声音喊到底、一个步调抓到底，确保政府工作始终沿着正确方向前进。

（二）严抓廉政建设，扎紧制度笼子。坚决落实全面从严治党主体责任，加强政府系统党建工作，严格“三会一课”制度，严肃党内政治生活，打造坚强战斗堡垒。深入开展党风廉政建设和反腐败工作，坚定不移贯彻中央八项规定精神，驰而不息纠“四风”、树新风，让廉洁从政成为政府工作的鲜明底色。加大审计监督力度，从严查处侵害群众利益的行为，严防严控重点工程和重点领域廉政风险，始终保持惩治腐败“零容忍”高压态势，营造风清气正政治生态。

（三）紧抓能力建设，练就过硬本领。围绕增强“八种本领”、做到“五个过硬”，进一步提升贯彻执行能力，强化“钉钉子”精神，坚持“日清日结”制度，今日事、今日毕，当好抓落实的“急先锋”；进一步提升攻坚克难能力，坚持理论学习、业务学习制度，大兴调查研究之风，奔着问题去、带着成果回，在担当实干中磨练扛事成事的宽肩膀、真本领；进一步提升创新竞进能力，鼓励支持广大党员干部解放思想、探索实践，争做行业排头兵、发展领头羊。

（四）常抓法治建设，坚持依法行政。深入贯彻全面依法治国基本方略，带头普法学法、守法用法，依法依规依程序履行政府职能，切实做到不失位不越位不缺位。严格执行专家论证、风险评估、合法性审查等重大行政决策法定程序，推进科学民主决策。自觉接受人大法律监督、工作监督和政协民主监督，广泛听取民主党派、工商联、无党派人士、人民团体意见，让人民监督权力，让权力在阳光下运行。

各位代表，习近平总书记指出，当今世界面临百年未有之大变局，变局中危和机同生并存，这给中华民族伟大复兴带来重大机遇。40年改革开放，沧州人民勠力同心、拼搏奋进，创造了无数骄人业绩，在前进的道路上，我们也同样会把握机遇，迎难而上，再创佳绩。让我们紧密团结在以习近平同志为核心的党中央周围，在中共沧州市委的坚强领导下，高举习近平新时代中国特色社会主义思想伟大旗帜，深入贯彻落实党的十九大精神，锐意进取、真抓实干，为加快建设新时代创新驱动经济强市、生态宜居美丽沧州作出新的更大贡献，以优异成绩庆祝中华人民共和国成立70周年！
